# Characteristics of Serum Lipid Metabolism among Women Complicated with Hypertensive Disorders in Pregnancy: A Retrospective Cohort Study in Mainland China

**DOI:** 10.1155/2024/9070748

**Published:** 2024-02-14

**Authors:** Lidong Liu, Xiaolei Zhang, Kaizhou Qin, Chengjie Xu, Fangyi Ruan, Yadan Liu, Huanqiang Zhao, Yinan Wang, Yu Xiong, Qiongjie Zhou, Xiaotian Li

**Affiliations:** ^1^Obstetrics and Gynecology Hospital of Fudan University, Fangxie Road 419, Huangpu, Shanghai, China; ^2^Department of Obstetrics and Gynecology, The First Affiliated Hospital of Shandong First Medical University, Jinan, Shandong, China; ^3^Shenzhen Maternity and Child Healthcare Hospital, Shenzhen, Guangdong, China

## Abstract

**Background:**

Altered maternal serum lipid metabolism is associated with hypertensive disorders in pregnancy (HDP). However, its range in pregnancy and characteristic among different subgroups of HDPs are unclear.

**Methods:**

Pregnant women with HDP who underwent antenatal care and delivered in Obstetrics and Gynecology Hospital of Fudan University during January 2018 to August 2022 were enrolled. The levels of total cholesterol (TC), triglycerides (TG), high-density lipoprotein cholesterol (HDLC), low-density lipoprotein cholesterol (LDLC), apolipoprotein (Apo)-A, B, and E, free fatty acids (FFA), and small and dense low-density lipoprotein cholesterol (sdLDL) were measured during 4–16 weeks and 28–42 weeks of pregnancy.

**Results:**

A total of 2648 pregnant women were diagnosed with HDP, 1,880 of whom were enrolled for final analysis, including 983 (52.3%) preeclampsia (PE), 676 (36.0%) gestational hypertension (GH), and 221 (11.7%) chronic hypertension (CH). For all HDPs, serum TC, TG, LDLC, HDLC, Apo-A, Apo-B, Apo-E, and sdLDL increased significantly during pregnancy, while FFA decreased significantly. Notably, the levels of TC, LDLC, Apo-B, and sdLDL in PE group were equal to or lower than those in CH group at 4–16 weeks of pregnancy, but increased greatly during pregnancy (*P* < 0.05).

**Conclusions:**

Maternal serum lipid levels changed through pregnancy among women with HDPs. Women complicated with PE seem to have undergone a more significant serum lipid change compared to those with GH or CH.

## 1. Introduction

Hypertensive disorders in pregnancy (HDP) are a pregnancy-specific syndrome, with a 2–8% incidence [1, 2]. There were evidences suggesting that hyperlipidemia and related metabolic disorders be associated with higher risk of HDP [3–5]. A meta-analysis of 74 studies [6] showed that total cholesterol and triglyceride levels were elevated in women with preeclampsia during the third trimester of pregnancy. Lipid metabolism during pregnancy is the adaptive increases in serum total cholesterol and triglyceride levels with the increasing level of estrogen, progesterone, and lactogen during pregnancy, providing a fatty acid energy bank for fetal growth and placental tissue steroid synthesis in late pregnancy [7]. Till now, the changing ranges of lipid metabolism among women complicated with HDP spanning the whole pregnancy are lacked.

Moreover, the pathogenesis of different subgroups of HDP, such as preeclampsia (PE), gestational hypertension (GH), and chronic hypertension (CH), is considered varied in oxidative stress and endothelial dysfunction [8], which is closely related with metabolism of lipoproteins [9, 10]. An important gap is the characteristics of maternal lipid metabolism among these HDPs. Thus, we hypothesize that maternal lipid metabolism may differ among subgroups of HDPs.

Herein, this retrospective cohort study aimed to describe the changes in serum lipid metabolism among women complicated with PE, GH, and CH at 4–16 weeks and 28–42 weeks of pregnancy.

## 2. Materials and Methods

### 2.1. Study Design and Participants

A retrospective cohort study was conducted in Obstetrics and Gynecology Hospital of Fudan University. Pregnant women with HDP who underwent antenatal care and delivered from January 2018 to August 2022 were included. Those women with multiple pregnancies, maternal complications including cardiovascular disease, diabetes, cancer, kidney, and liver disease, and incomplete or missing medical records were excluded.

### 2.2. Data Collection

Maternal baseline and clinical information were extracted from medical records. Baseline information included maternal age, weight, height, educational level, ABO and Rh blood types, histories of preeclampsia, alcohol consumption, and smoking. Prepregnancy weight, alcohol consumption, and smoking information was self-reported at the first antenatal visit. Educational levels were classified as low (high school or below) and high (bachelor or above).

Related clinical information was picked from the participants delivery record, including systolic and diastolic blood pressure (BP) at admission(The same arm of the pregnant woman should be measured at least twice, and the interval between the two measurements should be ≥4 h), proteinuria (protein excretion in a 24 h urine collection), antiphospholipid syndrome (APS) [11], gestational diabetes mellitus (GDM)(any one of fasting blood glucose ≥5.1 mmol/L, 1 h blood glucose ≥10.0 mmol/L, or 2 h blood glucose ≥8.5 mmol/L at 24 to 28 weeks of gestation by the 75 g oral glucose tolerance test (OGTT)) [12], postpartum hemorrhage [13], placental abruption [14], gestational age of delivery, mode of delivery (divided into vaginal delivery and cesarean section), placental weight, neonatal birthweight, sex, and distress. Apgar score [15] includes appearance, pulse, grimace, activity, and respiration. Low Apgar score is defined as ≤7.

HDPs included PE, GH, and CH [16]. PE was defined as elevated blood pressure (systolic BP ≥ 140 mmHg or diastolic BP ≥ 90 mmHg), with proteinuria (>0.3 g/24 h) or other organ dysfunctions after 20 weeks gestation [17]. Severe PE refers to PE with severe features, including higher blood pressure (systolic BP ≥ 160 mmHg or diastolic BP ≥ 110 mmHg), lower platelet count (<100,000 × 10^9^/L), impaired organ function or neurological symptoms [18]. GH was defined as newly onset hypertension after 20 weeks of gestation, with previously normal blood pressure and without proteinuria [1, 18]. CH was defined as chronic hypertension diagnosed before 20 weeks of gestation [1, 19].

### 2.3. Serum Lipid Measures

The levels of fasting total cholesterol (TC), triglycerides (TG), high-density lipoprotein cholesterol (HDLC), low-density lipoprotein cholesterol (LDLC), apolipoprotein (Apo)-A, B, and E, free fatty acids (FFA), and small and dense low-density lipoprotein cholesterol (sdLDL) were measured during 4–16 weeks and 28–42 weeks of pregnancy. A total of 2 mL of peripheral venous blood was collected routinely at 4–16 weeks and 28–42 weeks of pregnancy after 8 hours of fasting during outpatient and hospitalization, by trained nurses. Serum samples were tested within 2 hours and recorded by two independent staff. Automatic biochemical analyzer (LABOSPECT 008*α*, Japan) was used to detect the levels of blood lipids, including TC (LabAssay cholesterol, Wako Pure Chemical Co. Ltd., Osaka, Japan), TG (LabAssay triglyceride, Wako), HDLC (HDL-cholesterol, Sekisui), LDLC (cholesterol LDL, Sekisui Medical technology Co., LTD., Tokyo, Japan), Apo-A (APO A AUTO·N (DAIICHI), Sekisui), Apo-B (APO B AUTO·N (DAIICHI), Sekisui), Apo-E (APO E AUTO·N (DAIICHI), Sekisui), FFA (NEFA FS kit, DiaSys Diagnostic Systems GmbH, Holzheim, Germany), and sdLDL (Zhejiang Dongou Diagnostics Co., Ltd., Wenzhou, China).

### 2.4. Statistical Analyses

Descriptive data were presented as mean ± SD (standard deviation) for continuous variables or number (%) for categorical variables. The range and percentiles of the maternal serum lipid concentration at 4–16 weeks and 28–42 weeks of pregnancy were described from 2.5% to 97.5%.

The means between 4-16 weeks and 28–42 weeks of pregnancy were compared by the paired Student's *t*-test. Analysis of variance (ANOVA) with Bonferroni test was used to determine statistical differences in the distribution of maternal serum lipid concentration and comparisons among PE, GH, and CH groups. SPSS (Statistical Package for the Social Sciences, Chicago, IL, USA) version 26.0 was used for statistical analysis. A *P* value < 0.05 was considered statistically significant.

## 3. Results

### 3.1. Enrollment of Participants

**Figure 1:**
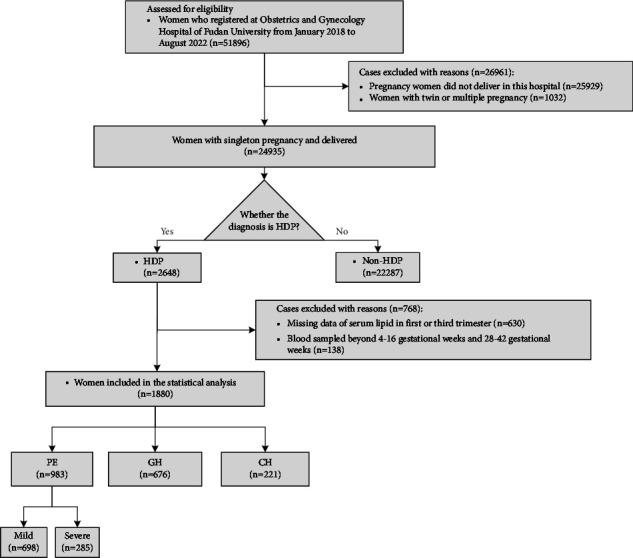
Flowchart of participant enrollment.

A total of 51,896 pregnant women registered at Obstetrics and Gynecology Hospital of Fudan University during January 2018 to August 2022, among whom 25,929 women were excluded due to nondelivery in our hospital, 1,032 women had twin or multiple pregnancies, and 22,287 women did not have HDP. Among 2,648 women complicated with HDPs, missing data of serum lipid at 4–16 weeks and 28–42 weeks of pregnancy (*n* = 630), serum lipid sampled beyond 4–16 gestational weeks and 28–42 gestational weeks (*n* = 138), were excluded. A total of 1,880 HDP women were enrolled for final analysis, including 983 (52.3%) PE, 676 (36.0%) GH, and 221 (11.7%) CH (Figure 1).

Maternal demographic characteristics were demonstrated in Table 1. As expected, higher proteinuria (1.34 ± 1.96 g vs. 0.41 ± 0.84 g vs. 0.65 ± 1.13 g), neonatal distress (1.93% vs. 0.30% vs. 0.90%), postpartum hemorrhage incidence (7.83% vs. 4.59% vs. 3.62%), and lower Apgar's score at 1 min (2.75% vs. 1.04% vs. 2.71%) were observed in women of PE among three groups. Also, compared with those women with PE and GH, women complicated with CH were of elder age (32.72 ± 4.34 years vs. 32.68 ± 4.46 years vs. 34.02 ± 4.78 years), having higher prepregnancy body mass index (BMI) (23.62 ± 3.95 kg/m^2^ vs. 23.38 ± 3.91 kg/m^2^ vs. 26.38 ± 5.55 kg/m^2^) and antenatal BMI (29.08 ± 3.88 kg/m^2^ vs. 28.58 ± 3.96 kg/m^2^ vs. 30.17 ± 5.00 kg/m^2^), earlier delivery age (38.17 ± 1.91 weeks vs. 39.00 ± 1.52 weeks vs. 37.53 ± 1.89 weeks), higher incidence of GDM rate (15.97% vs. 18.05% vs. 29.41%) and cesarean section rate (52.80% vs. 43.49% vs. 63.35%), lower incidence of postpartum hemorrhage rate (7.83% vs. 4.59% vs. 3.62%), and lower birthweight (3122.02 ± 610.06 g vs. 3271.73 ± 482.88 g vs. 2993.65 ± 574.10 g).

### 3.2. Maternal Serum Lipid Concentration at 4–16 weeks and 28–42 weeks of Pregnancy for All HDPs

During the whole pregnancy, serum lipid concentration significantly altered (Table 2). The levels of TC, TG, LDLC, HDLC, Apo-A, Apo-B, Apo-E, and sdLDL increased significantly from 4–16 weeks to 28–42 weeks of pregnancy (TC: 1.91 ± 1.21 mmol/L, TG: 3.39 ± 2.25 mmol/L, LDLC: 1.01 ± 0.97 mmol/L, HDLC: 0.30 ± 0.42 mmol/L, Apo-A: 0.43 ± 0.34 g/L, Apo-B: 0.49 ±0.27 g/L, Apo-E: 34.84 ± 29.18 g/L, and sdLDL: 0.56 ± 0.46 mmol/L, *P* < 0.001), while FFA decreased significantly (FFA: −0.12 ± 0.30 mmol/L, *P* < 0.001).

### 3.3. Maternal Serum Lipid Concentration in among Women with PE, GH, and CH

**Figure 2:**
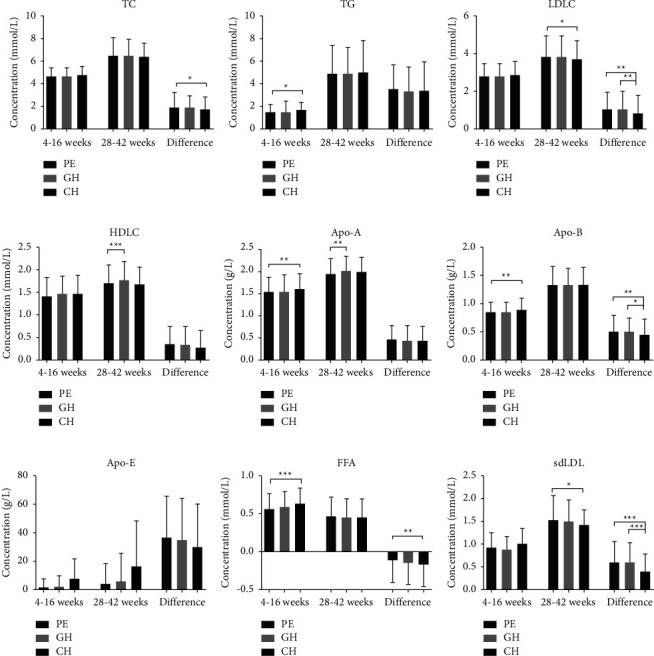
Comparison of maternal serum lipid concentrations among women with PE, GH, and CH.

The characteristics and validity number of serum lipid concentrations among three subgroups of HDPs was shown in Figure 2 and Table 3. Except for FAA of CH group (*P*=0.077), other lipid indicators have significant changes during pregnancy (*P* < 0.001). Notably, serum lipid levels among women with PE were significantly altered during the whole pregnancy, compared to those with GH and CH shown in Figure 2 and Table 3.

Higher levels of TC, LDLC, Apo-B, and sdLDL and lower levels of HDLC and Apo-A levels (*P* < 0.05) were found among women with PE than those with GH (*P* > 0.05), both at 4–16 weeks and 28–42 weeks of pregnancy. Notably, the increase in TC, LDLC, Apo-B, and sdLDL were greater in PE than in GH, and the decrease in HDLC and Apo-A were also greater in PE than in GH. Also, compared to women with CH, women with PE had lower level of TC, LDLC, HDLC, Apo-E, sdLDL (*P* > 0.05), TG, Apo-A, Apo-B, and FFA (*P* < 0.05) at 4–16 weeks of pregnancy; moreover, the increase in TC, LDLC, Apo-B, and sdLDL from 4-16 weeks to 28–42 weeks of pregnancy were significantly higher (*P* < 0.05) as shown in Figure 2 and Table 3.

Blood lipid differences between severe and mild preeclampsia in the PE group were analyzed as shown in Table S3. Our data showed that there was no statistical difference of blood lipid levels between mild and severe preeclampsia, except for Apo-A (*P* < 0.05).

## 4. Discussion

### 4.1. Main Finding

Altered lipid metabolism is associated with hypertensive disorders during pregnancy, but the characteristics during whole pregnancy among different subgroups of HDPs are unclear. Herein, we have demonstrated significant fluctuations of serum lipid levels among women complicated with HDPs, from 4-16 weeks to 28–42 weeks of pregnancy, characterized with significant difference among PE, GH, and CH.

### 4.2. Clinical Implications

HDP is a heterogeneous disease, with different types having different mechanisms and different effects on lipid metabolism [20, 21]. Our results showed that there were differences in lipid levels among PE, GH, and CH groups. TG, LDLC, Apo-B, and Apo-E levels in PE group increased greater than those in GH group, while HDLC and Apo-A levels decreased significantly from 4–16 weeks to 28–42 weeks of pregnancy, suggesting that abnormal lipid metabolism was more common in PE. The comparative data of PE group and CH group showed that the levels of TC, LDLC, Apo-B, and sdLDL were lower in PE group than those in CH group at 4–16 weeks of pregnancy, but increased more significantly during pregnancy, suggesting that the degree of these “bad” lipids increase during the course of pregnancy might play a more important role in the pathogenesis of PE.

Previous literature focused on the association of blood lipid comparison with pregnancy outcome between HDP and PE with normal pregnancy [3, 22, 23]. Thus, there was a lack of research on comparing serum lipids among different subtypes of HDP, and no consistent view on the change of serum lipids levels. Some studies [6, 10, 24] had shown that serum concentrations of TC, TG, LDLC, apolipoprotein, and FFA were higher in women with preeclampsia compared to controls with normal blood pressure, while other studies [25, 26] had shown that they were unchanged. A large prospective longitudinal cohort study [3] evaluated lipid elevation throughout pregnancy, showing that greater increase in TG levels from 4–16 weeks to 28–42 weeks of pregnancy were associated with an increased risk of HDP. A case-control study [27] included 1366 preeclampsia cases which also reported that elevated TG and Apo-E levels increased the risk of preeclampsia. In a review of 22 studies, Ray et al. [4] found that women with elevated TG had a 4 times greater risk of preeclampsia than women with normal TG. A meta-review of 74 studies [6] reported that preeclamptic women had elevated total cholesterol and triglyceride and lower HDLC levels than those of normotensive pregnant women at 28–42 weeks of pregnancy. Consistent with these findings, our results showed that serum lipids of HDP pregnant women were increased during pregnancy generally, except that FFA was decreased.

In addition, in terms of mechanism, it has been reported that GH was commonly seen in women with unfavorable metabolic profile, while PE was partly caused by genetic factors [28, 29]. At odds with them, our data of 1,880 HDP women showed that PE had the largest range of lipid metabolism fluctuation among GH, CH, and PE. Although all serum lipids of CH group were the highest at 4–16 weeks of pregnancy, the TC, sdLDL, LDLC, and Apo-B of PE group were the highest at 28–42 weeks of pregnancy and increased the most during pregnancy. Our results indicate that the increase, instead of the primary level, of “bad” lipids may play a more important role in the development of PE.

In addition, a higher rate of postpartum hemorrhage and placental abruption was noted in PE group. Along with the abundant evidence of endothelial progenitor cells [30] and sFlt-1 (soluble fms-like tyrosine kinase 1) and PlGF (placental growth factor) [31] in the placental dysfunction, our finding suggested potentially synergetic pathology of lipid alternation in postpartum hemorrhage and placental abruption among preeclamptic women. Detailed molecular mechanism is required to be further elucidated.

### 4.3. Strengths and Limitations

This was a large retrospective cohort study. As far as we know, we are the first to describe the range of HDP maternal serum lipids during pregnancy in the Chinese population and the effects of different types on lipids.

This study also had some limitations. First, since this was a single-center study which could not well represent the characteristics of the entire Chinese or Asian population, the multicenter and prospective study with a larger sample size would be more representative. Second, the confounding variables was not adjusted and recall bias could not be ruled out. For example, BMI and diet before pregnancy were important factors affecting dyslipidemia during pregnancy. Third, previous studies showed that serum lipids generally increase in pregnancy in both normotensive or hypertensive pregnancies [32, 33]. The relationship between increased ‘bad' lipids and preeclampsia needs to be further analyzed along with normotensive pregnancies.

## 5. Conclusions

In conclusion, maternal serum lipid metabolism is altered from 4–16 weeks to 28–42 weeks of pregnancy among women with HDPs. Women complicated with PE seem to undergo a more significant serum lipid change compared to those with GH and CH.

## Figures and Tables

**Table 1:** Baseline and clinical characteristics of enrolled women with HDPs.

Parameters	Total HDPs (*n* = 1880)	PE (*n* = 983)	GH (*n* = 676)	CH (*n* = 221)	*P*
Age (years), mean ± SD	32.86 ± 4.45	32.72 ± 4.34	32.68 ± 4.46	34.02 ± 4.78	<0.001
Prepregnancy BMI (kg/m^2^)^a^, mean ± SD	23.85 ± 4.25	23.62 ± 3.95	23.38 ± 3.91	26.38 ± 5.55	<0.001
Antenatal BMI (kg/m^2^)^b^, mean ± SD	29.03 ± 4.08	29.08 ± 3.88	28.58 ± 3.96	30.17 ± 5.00	<0.001
Educational level, *n* (%)					<0.001
High (bachelor or above)	552 (29.36)	254 (25.84)	225 (33.28)	73 (33.03)	
Low (high school or below)	154 (8.19)	74 (7.53)	49 (7.25)	31 (14.03)	
Unknown	1174 (62.45)	655 (66.63)	402 (59.47)	117 (52.94)	
ABO, *n* (%)					0.388
A	495 (26.33)	272 (27.67)	169 (25.00)	54 (24.43)	
AB	138 (7.34)	79 (8.04)	43 (6.36)	16 (7.24)	
B	459 (24.42)	222 (22.58)	177 (26.18)	60 (27.15)	
O	489 (26.01)	247 (25.13)	179 (26.48)	63 (28.51)	
Unknown	299 (15.90)	163 (16.58)	108 (15.98)	28 (12.67)	
Rh, *n* (%)					0.007
Rh (−)	11 (0.59)	3 (0.31)	3 (0.44)	5 (2.26)	
Rh (+)	1598 (85.00)	840 (85.45)	568 (84.03)	190 (85.97)	
Unknown	271 (14.41)	140 (14.24)	105 (15.53)	26 (11.77)	
History of PE, *n* (%)					0.008
Ex-PE	14 (0.75)	5 (0.51)	3 (0.44)	6 (2.71)	
Non-PE	1862 (99.04)	976 (99.29)	671 (99.26)	215 (97.29)	
Unknown	4 (0.21)	2 (0.20)	2 (0.30)	0 (0.00)	
History of alcohol, *n* (%)					0.091
Nondrinker	1874 (99.68)	979 (99.59)	676 (100.00)	219 (99.10)	
Drinker	6 (0.32)	4 (0.41)	0 (0.00)	2 (0.90)	
Smoking history, *n* (%)					0.091
Nonsmoker	1874 (99.68)	979 (99.59)	676 (100.00)	219 (99.10)	
Smoker	6 (0.32)	4 (0.41)	0 (0.00)	2 (0.90)	
Delivery history, *n* (%)					0.421
No	102 (5.43)	54 (5.49)	40 (5.92)	8 (3.62)	
Yes	1778 (94.57)	929 (94.51)	636 (94.08)	213 (96.38)	
Systolic BP (mmHg)^c^, mean ± SD	163.78 ± 16.89	164.90 ± 16.09	160.89 ± 17.50	166.05 ± 18.64	0.356
Diastolic BP (mmHg)^d^, mean ± SD	102.00 ± 11.09	101.78 ± 9.16	100.91 ± 13.05	105.63 ± 13.19	0.285
Proteinuria (g/24 h)^e^, mean ± SD	1.02 ± 1.71	1.34 ± 1.96	0.41 ± 0.84	0.65 ± 1.13	<0.001
APS, *n* (%)					0.146
No	1870 (99.47)	980 (99.69)	672 (99.41)	218 (98.64)	
Yes	10 (0.53)	3 (0.31)	4 (0.59)	3 (1.36)	
GDM, *n* (%)					<0.001
No	1536 (81.70)	826 (84.03)	554 (81.95)	156 (70.59)	
Yes	344 (18.30)	157 (15.97)	122 (18.05)	65 (29.41)	
Postpartum hemorrhage, *n* (%)					0.006
No	1764 (93.83)	906 (92.17)	645 (95.41)	213 (96.38)	
Yes	116 (6.17)	77 (7.83)	31 (4.59)	8 (3.62)	
Placental abruption, *n* (%)					0.145
No	1862 (99.04)	970 (98.68)	671 (99.26)	221 (100.00)	
Yes	18 (0.96)	13 (1.32)	5 (0.74)	0 (0.00)	
Mode of delivery, *n* (%)					<0.001
Transvaginal delivery	927 (49.31)	464 (47.20)	382 (56.51)	81 (36.65)	
Cesarean section	953 (50.69)	519 (52.80)	294 (43.49)	140 (63.35)	
Weeks of gestation at delivery (weeks)^f^, mean ± SD	38.37 ± 1.85	38.17 ± 1.91	39.00 ± 1.52	37.53 ± 1.89	<0.001
Placental weight (g)^g^, mean ± SD	514.66 ± 148.02	513.53 ± 157.57	523.71 ± 143.71	495.51 ± 121.30	0.121
Newborn birth length (cm)^h^, mean ± SD	49.33 ± 2.80	49.19 ± 2.47	49.70 ± 2.59	48.90 ± 4.15	0.001
Newborn birthweight (g)^i^, mean ± SD	3160.67 ± 570.53	3122.02 ± 610.06	3271.73 ± 482.88	2993.65 ± 574.10	<0.001
Newborn sex, *n* (%)					0.257
Female	941 (50.05)	504 (51.27)	324 (47.93)	113 (51.13)	
Male	937 (49.84)	479 (48.73)	350 (51.77)	108 (48.87)	
Unknown	2 (0.11)	0 (0.00)	2 (0.30)	0 (0.00)	
Apgar score-1 min, *n* (%)					0.002
≤7	40 (2.13)	27 (2.75)	7 (1.03)	6 (2.71)	
8-9	1371 (72.92)	707 (71.92)	484 (71.60)	180 (81.45)	
Unknown	469 (24.95)	249 (25.33)	185 (27.37)	35 (15.84)	
Apgar score-5 min, *n* (%)					0.012
≤7	5 (0.27)	3 (0.31)	1 (0.15)	1 (0.45)	
8-9	1405 (74.73)	731 (74.36)	489 (72.34)	185 (83.71)	
Unknown	470 (25.00)	249 (25.33)	186 (27.51)	35 (15.84)	
Neonatal distress, *n* (%)					0.011
No	1857 (98.78)	964 (98.07)	674 (99.70)	219 (99.10)	
Yes	23 (1.22)	19 (1.93)	2 (0.30)	2 (0.90)	

Data were expressed as mean ± standard deviation (SD) or count *N* (%). *P* < 0.05 was considered statistically significant. HDP: hypertensive disorder of pregnancy, PE: preeclampsia, GH: gestational hypertension, CH: chronic hypertension, BMI: body mass index, Rh: rhesus macacus, BP: blood pressure, APS: antiphospholipid syndrome, GDM: gestational diabetes mellitus, and Apgar: activity, pulse, grimace, appearance, and respiration. a: *n* = 1863, missing data for *n* = 17; b: *n* = 1803, missing data for *n* = 77; c: *n* = 148, missing data for *n* = 1732; d: *n* = 148, missing data for *n* = 1732; e: *n* = 1115, missing data for *n* = 765; f: *n* = 979, missing data for *n* = 901; g: *n* = 1118, missing data for *n* = 762; h: *n* = 1288, missing data for *n* = 592; i: *n* = 1872, missing data for *n* = 8.

**Table 2:** Maternal serum lipid concentration at 4–16 weeks and 28–42 weeks of pregnancy.

Serum lipid	Gestational week (weeks)	Number		Range			Reference interval	Percentile (%)												
		Val	Del	Min	Mean ± SD	Max	Percentiles (2.5–97.5%)	2.5	5	10	20	30	40	50	60	70	80	90	95	97.5
TC (mmol/L)	4–16	1880	0	1.99	4.64 ± 0.80	12.50	3.26–6.41	3.26	3.46	3.71	4.01	4.21	4.40	4.59	4.78	4.99	5.25	5.60	5.96	6.41
	28–42	1879	1	2.18	6.47 ± 1.37	22.46	4.22–9.25	4.22	4.53	4.93	5.40	5.70	6.05	6.37	6.68	7.05	7.45	8.16	8.74	9.25
	Difference^a^	1880	0	−1.16	1.91 ± 1.21	19.74	−0.08–4.47	−0.08	0.21	0.54	0.95	1.29	1.53	1.80	2.07	2.38	2.78	3.31	3.86	4.47

TG (mmol/L)	4–16	1880	0	0.35	1.51 ± 0.81	16.16	0.63–3.26	0.63	0.69	0.79	0.95	1.09	1.22	1.36	1.51	1.68	1.92	2.39	2.76	3.26
	28–42	1876	4	0.94	4.28 ± 2.10	21.65	1.74–9.85	1.74	1.98	2.32	2.73	3.07	3.45	3.83	4.23	4.75	5.46	6.73	8.25	9.85
	Difference	1871	9	−2.76	3.39 ± 2.25	18.91	0.65–9.21	0.65	0.95	1.29	1.75	2.13	2.48	2.93	3.39	3.90	4.60	6.09	7.38	9.21

LDLC (mmol/L)	4–16	1877	3	0.65	2.84 ± 0.67	9.58	1.63–4.19	1.63	1.80	2.01	2.33	2.53	2.69	2.84	2.97	3.13	3.32	3.59	3.90	4.19
	28–42	1670	210	0.62	3.86 ± 1.09	13.38	1.81–6.02	1.81	2.15	2.56	3.04	3.30	3.55	3.81	4.04	4.34	4.72	5.21	5.62	6.02
	Difference	1574	306	−4.46	1.01 ± 0.97	6.52	−0.81–3.01	−0.81	−0.44	−0.15	0.21	0.51	0.74	0.98	1.22	1.46	1.74	2.18	2.65	3.01

HDLC (mmol/L)	4–16	1877	3	0.43	1.42 ± 0.41	3.34	0.77–2.28	0.77	0.84	0.93	1.05	1.14	1.25	1.37	1.48	1.63	1.79	1.97	2.15	2.28
	28–42	1670	210	0.34	1.73 ± 0.41	3.55	1.04–2.61	1.04	1.14	1.22	1.38	1.50	1.59	1.70	1.79	1.91	2.05	2.26	2.45	2.61
	Difference	1574	306	−1.10	0.30 ± 0.42	1.89	−0.46–1.22	−0.46	−0.33	−0.20	−0.06	0.06	0.17	0.28	0.39	0.48	0.62	0.81	1.03	1.22

Apo-A (g/L)	4–16	1869	11	0.72	1.55 ± 0.35	2.64	0.95–2.21	0.95	1.01	1.10	1.21	1.30	1.42	1.55	1.67	1.78	1.88	2.02	2.12	2.21
	28–42	1650	230	0.89	1.96 ± 0.34	2.99	1.31–2.61	1.31	1.40	1.51	1.66	1.79	1.89	1.99	2.06	2.16	2.25	2.38	2.51	2.61
	Difference	1567	313	−0.74	0.43 ± 0.34	1.83	−0.16–1.17	−0.16	−0.07	0.03	0.14	0.23	0.31	0.40	0.48	0.57	0.70	0.90	1.06	1.17

Apo-B (g/L)	4–16	1869	11	0.20	0.84 ± 0.19	2.43	0.52–1.23	0.52	0.56	0.62	0.68	0.73	0.78	0.82	0.87	0.92	0.98	1.09	1.16	1.23
	28–42	1650	230	0.33	1.30 ± 0.30	3.31	0.76–1.97	0.76	0.86	0.95	1.06	1.14	1.21	1.28	1.36	1.44	1.53	1.68	1.81	1.97
	Difference	1566	314	−0.34	0.49 ± 0.27	2.09	0.03–1.08	0.03	0.10	0.16	0.27	0.35	0.42	0.47	0.53	0.61	0.70	0.84	0.95	1.08

Apo-E (g/L)	4–16	658	1222	17.00	42.81 ± 14.45	142.00	23.00–80.00	23.00	26.00	29.00	32.00	34.00	37.00	40.00	43.00	47.00	52.00	60.00	68.05	80.00
	28–42	672	1208	20.00	72.07 ± 30.41	288.00	35.00–141.18	35.00	38.00	43.00	50.00	55.00	60.00	66.00	72.00	80.00	91.00	105.70	121.70	141.18
	Difference	550	1330	−26.00	34.84 ± 29.18	222.00	−4.23–103.45	−4.23	2.00	7.00	15.00	19.00	24.00	30.00	35.00	43.00	51.00	67.90	79.00	103.45

FFA (mmol/L)	4–16	1877	3	0.06	0.58 ± 0.20	1.95	0.24–1.02	0.24	0.29	0.33	0.41	0.47	0.52	0.57	0.62	0.67	0.74	0.84	0.92	1.02
	28–42	1656	224	0.07	0.49 ± 0.21	2.09	0.16–0.98	0.16	0.20	0.24	0.31	0.37	0.42	0.47	0.52	0.58	0.65	0.77	0.87	0.98
	Difference	1573	307	−1.79	−0.12 ± 0.30	1.28	−0.63–0.55	−0.63	−0.57	−0.46	−0.36	−0.27	−0.21	−0.14	−0.07	0.01	0.09	0.24	0.39	0.55

sdLDL (mmol/L)	4–16	628	1252	0.17	0.91 ± 0.32	2.07	0.37–1.63	0.37	0.45	0.51	0.64	0.74	0.81	0.89	0.97	1.06	1.17	1.33	1.48	1.63
	28–42	837	1043	0.11	1.51 ± 0.49	6.56	0.73–2.44	0.73	0.84	0.97	1.14	1.26	1.36	1.46	1.57	1.70	1.87	2.11	2.28	2.44
	Difference	528	1352	−0.60	0.56 ± 0.46	2.28	−0.23–1.46	−0.23	−0.11	−0.01	0.18	0.28	0.41	0.53	0.65	0.78	0.94	1.17	1.33	1.46

Data were expressed as mean ± standard deviation (SD). *P* < 0.05 was considered statistically significant. TC: total cholesterol, TG: triglyceride, LDLC: low-density lipoprotein cholesterol, HDL: high-density lipoprotein cholesterol, Apo: apolipoprotein, FFA: free fatty acid, sdLDL: small dense LDLC, difference^a^ represent blood lipid values at weeks 28–42 of gestation minus the values at weeks 4–16 of gestation, Min: minimum, Max: maximum, Del: deletion, and Val: validity.

**Table 3:** Comparison of maternal serum lipid concentrations among women with PE, GH, and CH.

Gestational week (weeks)	Serum lipid	Total HDPs	PE	GH	CH	*P*	
						GH vs. PE	CH vs.PE
4–16	TC (mmol/L)	4.64 ± 0.80	4.65 ± 0.77	4.61 ± 0.83	4.73 ± 0.83	0.963	0.557
	TG (mmol/L)	1.51 ± 0.81	1.49 ± 0.73	1.50 ± 0.96	1.66 ± 0.68	1.000	0.012
	LDLC (mmol/L)	2.84 ± 0.67	2.86 ± 0.63	2.79 ± 0.71	2.91 ± 0.69	0.107	0.916
	HDLC (mmol/L)	1.42 ± 0.41	1.40 ± 0.41	1.43 ± 0.40	1.45 ± 0.40	0.191	0.243
	Apo-A (g/L)	1.55 ± 0.35	1.53 ± 0.35	1.56 ± 0.35	1.61 ± 0.36	0.222	0.004
	Apo-B (g/L)	0.84 ± 0.19	0.84 ± 0.18	0.82 ± 0.20	0.89 ± 0.20	0.155	0.001
	Apo-E (g/L)	42.81 ± 14.45	42.31 ± 13.69	42.84 ± 15.48	44.26 ± 14.00	1.000	0.726
	FFA (mmol/L)	0.58 ± 0.20	0.57 ± 0.20	0.58 ± 0.21	0.64 ± 0.20	1.000	<0.001
	sdLDL (mmol/L)	0.91 ± 0.32	0.92 ± 0.31	0.86 ± 0.30	1.01 ± 0.35	0.080	0.063
28–42	TC (mmol/L)	6.47 ± 1.37	6.49 ± 1.46	6.49 ± 1.30	6.31 ± 1.16	1.000	0.226
	TG (mmol/L)	4.28 ± 2.10	4.35 ± 2.06	4.16 ± 2.05	4.37 ± 2.43	0.236	1.000
	LDLC (mmol/L)	3.86 ± 1.09	3.90 ± 1.08	3.87 ± 1.10	3.66 ± 1.03	1.000	0.013
	HDLC (mmol/L)	1.73 ± 0.41	1.70 ± 0.40	1.78 ± 0.42	1.70 ± 0.39	<0.001	1.000
	Apo-A (g/L)	1.96 ± 0.34	1.94 ± 0.34	2.00 ± 0.34	1.99 ± 0.32	0.002	0.102
	Apo-B (g/L)	1.30 ± 0.30	1.31 ± 0.30	1.30 ± 0.30	1.28 ± 0.26	0.818	0.636
	Apo-E (g/L)	72.07 ± 30.41	72.12 ± 29.54	71.45 ± 32.17	73.39 ± 29.21	1.000	1.000
	FFA (mmol/L)	0.49 ± 0.21	0.49 ± 0.21	0.49 ± 0.21	0.50 ± 0.24	1.000	1.000
	sdLDL (mmol/L)	1.51 ± 0.49	1.55 ± 0.53	1.50 ± 0.48	1.42 ± 0.36	0.454	0.021

Difference^a^	TC (mmol/L)	1.91 ± 1.21	1.94 ± 1.32	1.91 ± 1.06	1.73 ± 1.17	1.000	0.045
	TG (mmol/L)	3.39 ± 2.25	3.47 ± 2.22	3.28 ± 2.17	3.33 ± 2.58	0.295	1.000
	LDLC (mmol/L)	1.01 ± 0.97	1.05 ± 0.95	1.03 ± 0.98	0.78 ± 1.00	1.000	0.001
	HDLC (mmol/L)	0.30 ± 0.42	0.29 ± 0.43	0.32 ± 0.40	0.25 ± 0.39	0.699	0.539
	Apo-A (g/L)	0.43 ± 0.34	0.42 ± 0.35	0.45 ± 0.33	0.40 ± 0.34	0.623	1.000
	Apo-B (g/L)	0.49 ± 0.27	0.50 ± 0.28	0.49 ± 0.25	0.43 ± 0.29	1.000	0.003
	Apo-E (g/L)	34.84 ± 29.18	36.76 ± 29.17	34.40 ± 29.02	30.35 ± 29.35	1.000	0.228
	FFA (mmol/L)	−0.12 ± 0.30	−0.10 ± 0.30	−0.14 ± 0.29	−0.17 ± 0.28	0.096	0.008
	sdLDL (mmol/L)	0.56 ± 0.46	0.60 ± 0.47	0.60 ± 0.44	0.39 ± 0.42	1.000	0.001

Data were expressed as mean ± standard deviation (SD). *P* < 0.05 was considered statistically significant. HDP: hypertensive disorder of pregnancy, PE: preeclampsia, GH: gestational hypertension, CH: chronic hypertension, TC: total cholesterol, TG: triglyceride, LDLC: low-density lipoprotein cholesterol, HDL: high-density lipoprotein cholesterol, Apo: apolipoprotein, FFA: free fatty acid, sdLDL: small dense LDLC, difference^a^ represent blood lipid values at weeks 28–42 of gestation minus the values at weeks 4–16 of gestation, and vs.: versus.

## Data Availability

The datasets used and analyzed during the current study are available from the corresponding author on reasonable request.

## Abbreviations

Apgar: Activity, pulse, grimace, appearance, and respiration
Apo: Apolipoprotein
APS: Antiphospholipid syndrome
BMI: Body mass index
BP: Blood pressure
CH: Chronic hypertension
CI: Confidence interval
FFA: Free fatty acid
GDM: Gestational diabetes mellitus
GH: Gestational hypertension
HDL: High-density lipoprotein cholesterol
HDP: Hypertensive disorder of pregnancy
LDLC: Low-density lipoprotein cholesterol
OGTT: Oral glucose tolerance test
PE: Preeclampsia
Rh: Rhesus macacus
SD: Standard deviation
sdLDL: Small dense LDLC
SPSS: Statistical package for the social sciences
TC: Total cholesterol
TG: Triglyceride
vs.: Versus.

## Supplementary Materials

Supplementary Materials Table S1: validity and deletion number of serum lipid concentrations among women with PE, GH, and CH. Table S2: comparison between maternal serum lipids concentrations of 4–16 weeks and 28–42 weeks of pregnancy in different types of HDP. Table S3: mean of maternal serum lipid concentrations between mild and severe preeclampsia. .
