# Supplementary material for: Characteristics of Serum Lipid Metabolism among Women Complicated with Hypertensive Disorders in Pregnancy: A Retrospective Cohort Study in Mainland China
Source: Obstet Gynecol Int. 2024 Feb 14;2024:9070748. doi: 10.1155/2024/9070748 (PMC10881237; doi:10.1155/2024/9070748)
Supplement: Supplementary Materials — Table S1: validity and deletion number of serum lipid concentrations among women with PE, GH, and CH. Table S2: comparison between maternal serum lipids concentrations of 4–16 weeks and 28–42 weeks of pregnancy in different types of HDP. Table S3: mean of maternal serum lipid concentrations between mild and severe preeclampsia. . [file 9070748.f1.zip › Table S1 (1).docx]

**Table S1. Validity and deletion number of serum lipid concentrations among women with PE, GH and CH.**

|  |  | **Total HDPs** | | **PE** | | **GH** | | **CH** | |
| --- | --- | --- | --- | --- | --- | --- | --- | --- | --- |
| **Gestational**  **week**  **(weeks)** | **Serum lipid** | **Validity** | **Deletion** | **Validity** | **Deletion** | **Validity** | **Deletion** | **Validity** | **Deletion** |
| 4-16 | TC (mmol/L) | 1880 | 0 | 983 | 0 | 676 | 0 | 221 | 0 |
|  | TG (mmol/L) | 1880 | 0 | 983 | 0 | 676 | 0 | 221 | 0 |
|  | LDLC (mmol/L) | 1877 | 3 | 981 | 2 | 675 | 1 | 221 | 0 |
|  | HDLC (mmol/L) | 1877 | 3 | 981 | 2 | 675 | 1 | 221 | 0 |
|  | Apo-A(g/L) | 1869 | 11 | 975 | 8 | 675 | 1 | 219 | 2 |
|  | Apo-B(g/L) | 1869 | 11 | 975 | 8 | 675 | 1 | 219 | 2 |
|  | Apo-E(g/L) | 658 | 1222 | 303 | 680 | 255 | 421 | 100 | 121 |
|  | FFA (mmol/L) | 1877 | 3 | 981 | 2 | 675 | 1 | 221 | 0 |
|  | sdLDL(mmol/L) | 628 | 1252 | 273 | 710 | 251 | 425 | 104 | 117 |
| 28-42 | TC (mmol/L) | 1879 | 1 | 982 | 1 | 676 | 0 | 221 | 0 |
|  | TG (mmol/L) | 1876 | 4 | 981 | 2 | 674 | 2 | 221 | 0 |
|  | LDLC (mmol/L) | 1670 | 210 | 881 | 102 | 583 | 93 | 206 | 15 |
|  | HDLC (mmol/L) | 1670 | 210 | 881 | 102 | 583 | 93 | 206 | 15 |
|  | Apo-A(g/L) | 1650 | 230 | 873 | 110 | 576 | 100 | 201 | 20 |
|  | Apo-B(g/L) | 1650 | 230 | 873 | 110 | 576 | 100 | 201 | 20 |
|  | Apo-E(g/L) | 672 | 1208 | 333 | 650 | 239 | 437 | 100 | 121 |
|  | FFA (mmol/L) | 1656 | 224 | 874 | 109 | 579 | 97 | 203 | 18 |
|  | sdLDL(mmol/L) | 837 | 1043 | 407 | 576 | 301 | 375 | 129 | 92 |
| Difference^a^ | TC (mmol/L) | 1880 | 0 | 983 | 0 | 676 | 0 | 221 | 0 |
|  | TG (mmol/L) | 1871 | 9 | 979 | 4 | 671 | 5 | 221 | 0 |
|  | LDLC (mmol/L) | 1574 | 306 | 836 | 147 | 537 | 139 | 201 | 20 |
|  | HDLC (mmol/L) | 1574 | 306 | 836 | 147 | 537 | 139 | 201 | 20 |
|  | Apo-A(g/L) | 1567 | 313 | 830 | 153 | 537 | 139 | 200 | 21 |
|  | Apo-B(g/L) | 1566 | 314 | 830 | 153 | 536 | 140 | 200 | 21 |
|  | Apo-E(g/L) | 550 | 1330 | 254 | 729 | 208 | 468 | 88 | 133 |
|  | FFA (mmol/L) | 1573 | 307 | 836 | 147 | 536 | 140 | 201 | 20 |
|  | sdLDL(mmol/L) | 528 | 1352 | 234 | 749 | 205 | 471 | 89 | 132 |

PE: preeclampsia, GH: Gestational hypertension, CH: Chronic hypertension with superimposed preeclampsia, TC: Total Cholesterol, TG: Triglyceride, LDLC: Low-density lipoprotein cholesterol, HDL: High-density lipoprotein cholesterol, Apo: Apolipoprotein, FFA: Free fatty acid, sdLDL: small dense LDLC, Difference^a^: represent blood lipid values at weeks 28-42 of gestation minus the values at weeks 4-16 of gestation.
