# Supplementary material for: Characteristics of Serum Lipid Metabolism among Women Complicated with Hypertensive Disorders in Pregnancy: A Retrospective Cohort Study in Mainland China
Source: Obstet Gynecol Int. 2024 Feb 14;2024:9070748. doi: 10.1155/2024/9070748 (PMC10881237; doi:10.1155/2024/9070748)
Supplement: Supplementary Materials — Table S1: validity and deletion number of serum lipid concentrations among women with PE, GH, and CH. Table S2: comparison between maternal serum lipids concentrations of 4–16 weeks and 28–42 weeks of pregnancy in different types of HDP. Table S3: mean of maternal serum lipid concentrations between mild and severe preeclampsia. . [file 9070748.f1.zip › Table S2 (1).docx]

**Table S2. Comparison between maternal serum lipids concentrations of 4-16 weeks and 28-42 weeks of pregnancy in different types of HDP.**

|  | ***P*** | | | |
| --- | --- | --- | --- | --- |
| **Serum lipid** | **Total HDPs** | **PE** | **GH** | **CH** |
| TC (mmol/L) | <0.001 | <0.001 | <0.001 | <0.001 |
| TG (mmol/L) | <0.001 | <0.001 | <0.001 | <0.001 |
| LDLC (mmol/L) | <0.001 | <0.001 | <0.001 | <0.001 |
| HDLC (mmol/L) | <0.001 | <0.001 | <0.001 | <0.001 |
| Apo-A(g/L) | <0.001 | <0.001 | <0.001 | <0.001 |
| Apo-B(g/L) | <0.001 | <0.001 | <0.001 | <0.001 |
| Apo-E(g/L) | <0.001 | <0.001 | <0.001 | <0.001 |
| FFA (mmol/L) | <0.001 | <0.001 | <0.001 | 0.077 |
| sdLDL(mmol/L) | <0.001 | <0.001 | <0.001 | <0.001 |

*P* < 0.05 was considered statistically significant. HDP: Hypertensive disorder of pregnancy, PE: preeclampsia, GH: Gestational hypertension, CH: Chronic hypertension with superimposed preeclampsia, TC: Total Cholesterol, TG: Triglyceride, LDLC: Low-density lipoprotein cholesterol, HDL: High-density lipoprotein cholesterol, Apo: Apolipoprotein, FFA: Free fatty acid, sdLDL: small dense LDLC.
