# Supplementary material for: Characteristics of Serum Lipid Metabolism among Women Complicated with Hypertensive Disorders in Pregnancy: A Retrospective Cohort Study in Mainland China
Source: Obstet Gynecol Int. 2024 Feb 14;2024:9070748. doi: 10.1155/2024/9070748 (PMC10881237; doi:10.1155/2024/9070748)
Supplement: Supplementary Materials — Table S1: validity and deletion number of serum lipid concentrations among women with PE, GH, and CH. Table S2: comparison between maternal serum lipids concentrations of 4–16 weeks and 28–42 weeks of pregnancy in different types of HDP. Table S3: mean of maternal serum lipid concentrations between mild and severe preeclampsia. . [file 9070748.f1.zip › Table S3 (1).docx]

**Table S3. Mean of maternal serum lipid concentrations between mild and severe preeclampsia.**

|  |  | **PE** | | ***P*** |
| --- | --- | --- | --- | --- |
| **Gestational**  **week**  **(weeks)** | **Serum lipid** | **Mild** | **Severe** |  |
| 4-16 | TC (mmol/L) | 4.66±0.77 | 4.61±0.76 | 0.366 |
|  | TG (mmol/L) | 1.48±0.75 | 1.52±0.65 | 0.410 |
|  | LDLC (mmol/L) | 2.87±0.62 | 2.81±0.66 | 0.170 |
|  | HDLC (mmol/L) | 1.39±0.41 | 1.40±0.41 | 0.774 |
|  | Apo-A(g/L) | 1.52±0.35 | 1.54±0.36 | 0.369 |
|  | Apo-B(g/L) | 0.84±0.18 | 0.83±0.18 | 0.439 |
|  | Apo-E(g/L) | 42.56±14.06 | 41.60±12.68 | 0.591 |
|  | FFA(mmol/L) | 0.57±0.20 | 0.57±0.20 | 0.763 |
|  | sdLDL(mmol/L) | 0.93±0.29 | 0.91±0.34 | 0.589 |
| 28-42 | TC (mmol/L) | 6.55±1.48 | 6.36±1.38 | 0.055 |
|  | TG (mmol/L) | 4.33±2.07 | 4.38±2.04 | 0.746 |
|  | LDLC (mmol/L) | 3.95±1.10 | 3.79±1.04 | 0.056 |
|  | HDLC (mmol/L) | 1.71±0.40 | 1.66±0.39 | 0.118 |
|  | Apo-A(g/L) | 1.95±0.33 | 1.91±0.35 | 0.183 |
|  | Apo-B(g/L) | 1.32±0.30 | 1.29±0.30 | 0.108 |
|  | Apo-E(g/L) | 70.81±29.55 | 75.07±29.44 | 0.226 |
|  | FFA(mmol/L) | 0.49±0.21 | 0.50±0.23 | 0.680 |
|  | sdLDL(mmol/L) | 1.57±0.53 | 1.50±0.51 | 0.222 |
| Difference^a^ | TC (mmol/L) | 1.96±1.34 | 1.90±1.26 | 0.456 |
|  | TG (mmol/L) | 3.52±2.26 | 3.36±2.12 | 0.307 |
|  | LDLC (mmol/L) | 1.06±0.97 | 1.04±0.91 | 0.782 |
|  | HDLC (mmol/L) | 0.30±0.43 | 0.28±0.44 | 0.520 |
|  | Apo-A(g/L) | 0.44±0.35 | 0.38±0.35 | 0.040 |
|  | Apo-B(g/L) | 0.51±0.27 | 0.49±0.28 | 0.487 |
|  | Apo-E(g/L) | 36.41±29.42 | 37.71±28.69 | 0.752 |
|  | FFA(mmol/L) | -0.10±0.31 | -0.10±0.30 | 0.772 |
|  | sdLDL(mmol/L) | 0.60±0.46 | 0.58±0.49 | 0.814 |

Data were expressed as Mean±Standard deviation (SD). *P* < 0.05 was considered statistically significant. PE: preeclampsia, TC: Total Cholesterol, TG: Triglyceride, LDLC: Low-density lipoprotein cholesterol, HDL: High-density lipoprotein cholesterol, Apo: Apolipoprotein, FFA: Free fatty acid, sdLDL: small dense LDL-C, Difference^a^: represent blood lipid values at weeks 28-42 of gestation minus the values at weeks 4-16 of gestation.
